# Word Categorization of Vowel Durational Changes in Speech-Modulated Bone-Conducted Ultrasound

**DOI:** 10.3390/audiolres11030033

**Published:** 2021-07-14

**Authors:** Tadao Okayasu, Tadashi Nishimura, Akinori Yamashita, Yoshiki Nagatani, Takashi Inoue, Yuka Uratani, Toshiaki Yamanaka, Hiroshi Hosoi, Tadashi Kitahara

**Affiliations:** 1 Department of Otolaryngology-Head and Neck Surgery, Nara Medical University, 840 Shijo-cho, Kashihara 634-8522, Japan; t-nishim@naramed-u.ac.jp (T.N.); akinori@naramed-u.ac.jp (A.Y.); ysd811yk@yahoo.co.jp (Y.U.); toshya@naramed-u.ac.jp (T.Y.); tkitahara@naramed-u.ac.jp (T.K.); 2 Pixie Dust Technologies, 3F, 4F, Sumitomo Fudosan Suidobashi Nisiguchi Bldg, 2-20-5, Kanda-Misakicho, Chiyoda-ku, Tokyo 101-0061, Japan; yoshiki.nagatani@pixiedusttech.com; 3 Institute for Clinical and Translational Science, Nara Medical Univesity, 840 Shijo-cho, Kashihara 634-8522, Japan; tk-inoue@naramed-u.ac.jp; 4 MBT (Medicine-Based Town) Institute, Nara Medical University, 840 Shijo-cho, Kashihara 634-8522, Japan; hosoi@naramed-u.ac.jp

**Keywords:** bone-conduction, ultrasound, ultrasonic perception, prosody, amplitude modulation, vowel

## Abstract

Ultrasound can deliver speech information when it is amplitude-modulated with speech and presented via bone conduction. This speech-modulated bone-conducted ultrasound (SM-BCU) can also transmit prosodic information. However, there is insufficient research on the recognition of vowel duration in SM-BCU. The aim of this study was to investigate the categorization of vowel durational changes in SM-BCU using a behavioral test. Eight Japanese-speaking participants with normal hearing participated in a forced-choice behavioral task to discriminate between “hato” (pigeon) and “haato” (heart). Speech signal stimuli were presented in seven duration grades from 220 ms to 340 ms. The threshold at which 50% of responses were “haato” was calculated and compared for air-conducted audible sound (ACAS) and SM-BCU. The boundary width was also evaluated. Although the SM-BCU threshold (mean: 274.6 ms) was significantly longer than the ACAS threshold (mean: 269.6 ms), there were no differences in boundary width. These results suggest that SM-BCU can deliver prosodic information about vowel duration with a similar difference limen to that of ACAS in normal hearing.

## 1. Introduction

Ultrasound of frequencies higher than approximately 20–24 kHz [1] are not audible to humans via air-conduction. However, when it is presented via bone-conduction, humans can perceive ultrasound up to approximately 120 kHz [2] as an auditory sensation. This phenomenon was first reported by Gavreau in 1948 [3]. Several studies have identified the characteristics of ultrasonic perception. For example, the pitch of bone-conducted ultrasound (BCU) is similar to that of high frequency air-conducted audible sound (ACAS) (approximately 8–16 kHz) [2,4,5], but the just noticeable frequency difference is worse than that of ACAS [6,7]. BCU has a narrower dynamic range of loudness than ACAS [8,9] and is difficult to mask with ACAS [4]. An interesting characteristic of BCU is that some patients with profound hearing loss can perceive BCU as an auditory sensation [6,10,11]. There are several differences in the perceptual characteristics between BCU and ACAS.

The peripheral perceptual mechanism of BCU has been studied using electrophysiological examination. One study obtained the BCU-evoked action potential using electrocochleography in guinea pigs [12]. Several studies have investigated the central perceptual mechanism of BCU in humans using magnetoencephalography (MEG) and positron-emission tomography (PET) [13,14,15,16,17,18]. Responses evoked by BCU have been detected in the auditory cortex of both normal hearing and deaf individuals [10,11]. These objective observations demonstrate that BCU is perceived as an auditory sensation.

To clarify the peripheral perceptual mechanism for BCU, the masking produced by BCU and ACAS have been investigated [8,19]. Furthermore, the impact of cisplatin administration on the BCU threshold has been evaluated in patients with head and neck cancer [20]. The results of these studies indicate the following unique peripheral perceptual mechanism of BCU. BCU perception depends on inner hair cell activity induced by ultrasound, not on enhancement by outer hair cells in the basal turn of the cochlea [8,19,20,21]. However, further evidence is needed to confirm this mechanism.

Some patients with profound hearing loss can hear BCU, and speech-modulated (SM) BCU can deliver speech sounds [6]. These characteristics suggest that BCU hearing aids [22] and tinnitus treatments [23] could be developed for patients with profound hearing loss. The present BCU hearing aid enables normal hearing and profoundly deaf individuals to recognize 60–70% and approximately 30% of speech words, respectively [24,25,26,27]. Moreover, prosody is important for speech information such as questions or affirmations, and for emotional expression. We demonstrated that BCU can transmit prosodic information about pitch intonation [28]. One feature of prosody is vowel duration, which plays an important role in the determination of semantic meaning in Japanese. For example, “tori” and “toori” (short- and long-duration vowels) mean bird and street, respectively. However, there is insufficient research on prosodic information about vowel duration in BCU. The aim of the present study was to investigate the categorization of vowel durational changes in SM-BCU. Assessing the ability to discriminate vowel durational changes in SM-BCU is important for the clinical application of BCU hearing aids.

## 2. Materials and Methods

### 2.1. Participants

Participants were eight healthy volunteers with normal hearing (four women, four men; age range 22–36 years). Their thresholds as determined by conventional audiometry were 20 dB HL or lower. Participants provided written consent after receiving information about all experimental procedures and the study aim. All procedures were approved by the ethics committee of Nara Medical University.

### 2.2. Stimuli

**Figure 1:**
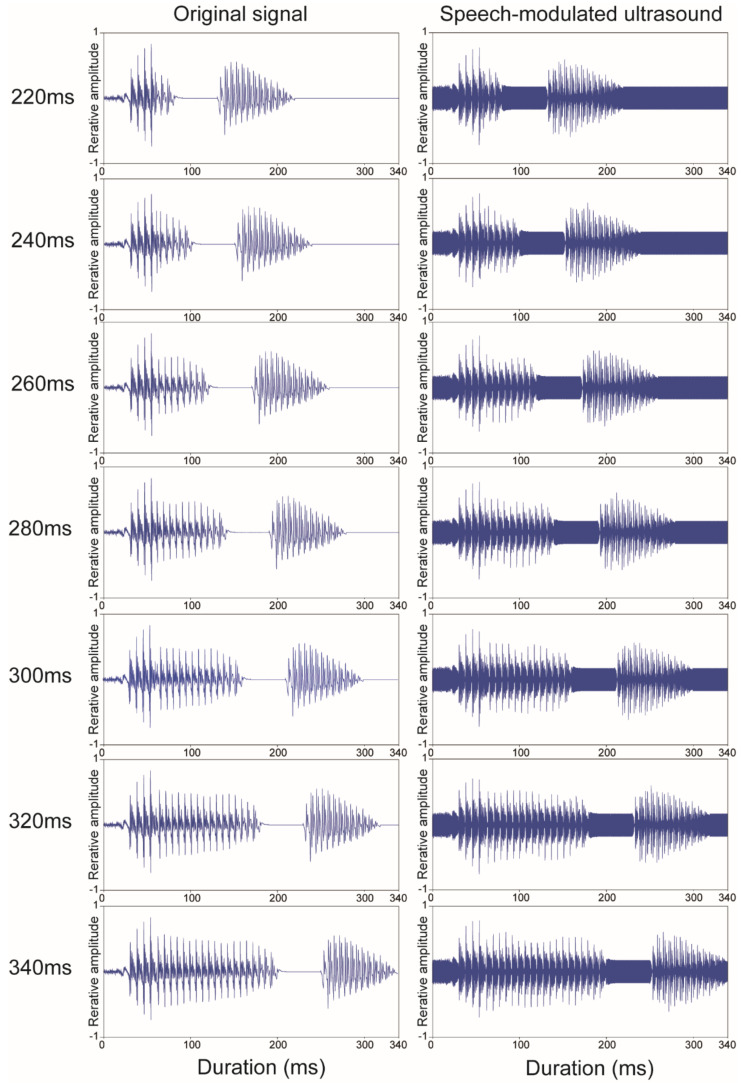
Speech signal waveforms of the original speech and the speech-modulated ultrasonic sounds.

The categorization of “hato” or “haato” was investigated. The Japanese word “hato” has a short-duration vowel and means pigeon. The Japanese word “haato” has a long-duration vowel and means heart. The words are differentiated by the duration of the vowel /a/. Stimuli were generated based on the speech signal “hato” recorded from a native adult male in an anechoic chamber. The shortest vowel duration of /a/ in “hato” (220 ms) was extended by seven grades in 20 ms steps to produce “haato,” which had the longest vowel duration (340 ms) (Figure 1). An analysis-by-synthesis system by Praat Software [29] was used to synthesize vowel duration. During editing, the same silent interval (40 ms) and syllable /to/ (90 ms) were spliced for all stimuli. The intensity and the vocal pitch contour (F0 contour) were kept constant across stimuli. The high frequency component (over 9 kHz) of the speech signal was eliminated using a low pass filter to prevent demodulation by amplitude modulation.

### 2.3. Discrimination Task

Participants performed a behavioral perceptual categorization task, in which they were forced to categorize stimuli as “hato” or “haato.” One session consisted of 10 stimuli with seven durational grades, from 220 ms to 340 ms, in random order. The stimulus interval was set at 2.0 s. Each participant performed for a total of 70-words per presentation. The ACAS experiment was administered first, followed by the SM-BCU experiment.

### 2.4. Procedure

The ACAS stimuli were presented with an earphone (SR-303; STAX, Miyoshi-machi, Japan) to the left ear. The SM-BCU stimuli were presented to the left mastoid by a ceramic vibrator developed for and used in our previous study [8].

Prior to the behavioral tests, ACAS and SM-BCU thresholds for the left ear were measured for each participant using tone bursts of 1000 Hz and 30 kHz, respectively. Their duration was set to 300 ms with 50 ms rise and fall ramps. The stimulus rate was 2 Hz. ACAS and ultrasound were generated using a function generator (WF1946; NF Electronic Instruments Co., Yokohama, Japan). Sound intensities were controlled using a programmable attenuator (PA5; Tucker-Davis Technologies, Gainesville, FL, USA) with 5.0 dB and 1.0 dB steps, respectively. The obtained thresholds were operationally defined as 0 dB sensation level (SL). The ACAS test stimuli were delivered to the left ear with an intensity of 40 dB SL. The SM-BCU intensity was set at 15 dB SL to take account of the narrow dynamic range of BCU [8]. These experiments were carried out in a soundproofed room.

In the SM-BCU test, the speech stimuli were modulated onto an ultrasonic carrier with a 30 kHz sine wave. Amplitude modulation was based on a double-sideband transmitted carrier with a modulation depth of 1.0. The modulated signal was calculated using the following formula:

U(t) = 1/2 × (1 + S(t)/Sc) × sin(2πfct)

where S(t) is the speech signal, Sc is the peak amplitude of the sinusoidal wave whose equivalent continuous A-weighted sound pressure level was equal to the speech signals, and fc is the carrier frequency (30 kHz). Figure 1 shows the waveforms of the signals.

### 2.5. Analysis

**Figure 2:**
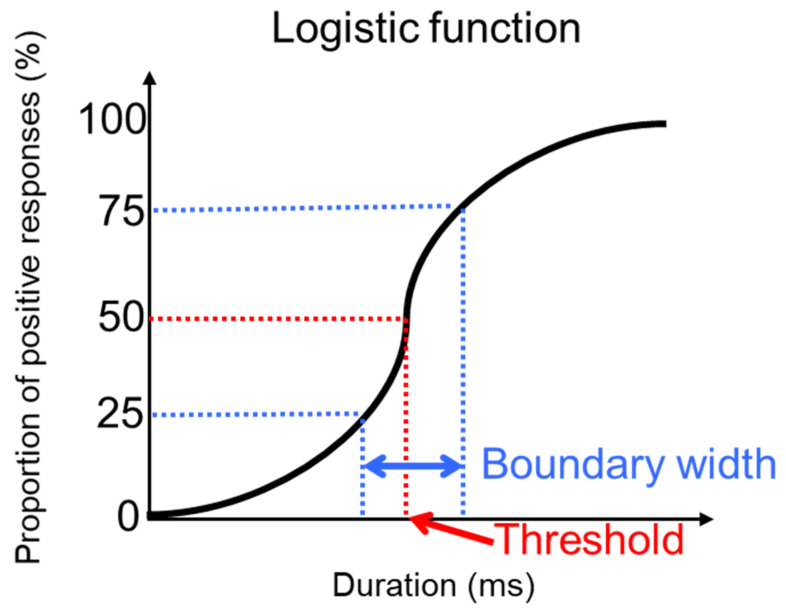
Evaluation of the categorization boundary using a logistic function.

To evaluate the categorization boundary, the relationship between the proportion of responses and the stimulus duration was approximated using a three-parameter logistic function. The stimulus duration at which 50% of responses were “haato” was defined as the threshold (Figure 2). The boundary width was defined as the stimulus duration at which 75% of responses were “haato” minus the stimulus duration at which 25% of responses were “haato” [30,31]. The threshold and the boundary width for ACAS and SM-BCU were calculated. These analyses were performed using JMP Pro version 15.2.1 (SAS Institute Inc., Cary, NC, USA).

### 2.6. Statistics

The threshold and boundary width were compared between ACAS and SM-BCU using the Wilcoxon matched-pairs signed rank test. These statistical analyses were performed using GraphPad software (GraphPad Prism version 7.02; GraphPad Software, Inc., LaJolla, CA, USA). Values of *p* < 0.05 were considered significant.

## 3. Results

**Figure 3:**
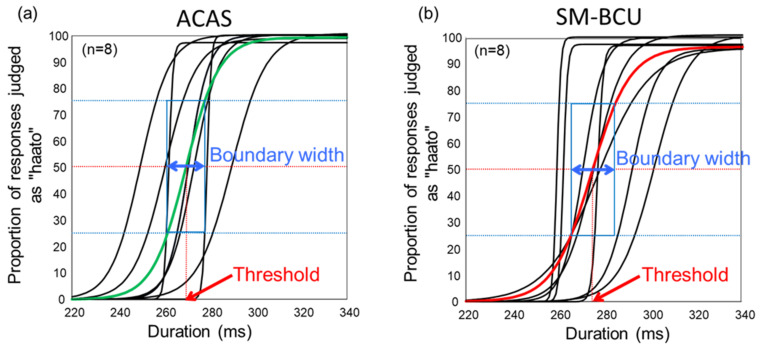
Logistic functions for air-conducted audible sound (ACAS) (**a**) and speech-modulated bone-conducted ultrasound (SM-BCU) (**b**).

**Figure 4:**
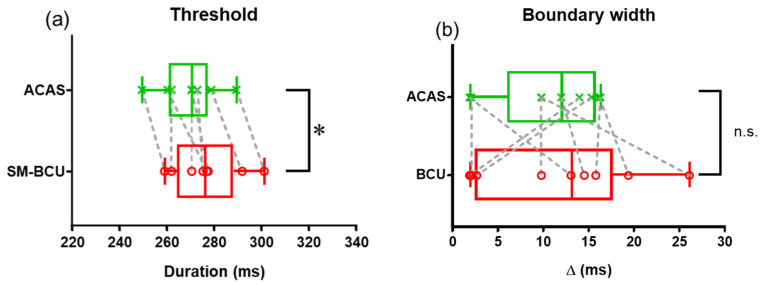
Threshold (**a**) and boundary width (**b**) for ACAS and SM-BCU. The asterisk indicates a statistically significant result from the Wilcoxon matched-pairs signed rank test (* *p* < 0.05). ACAS, air-conducted audible sound; SM-BCU, speech-modulated bone-conducted ultrasound.

Subjective perception of hearing for SM-BCU is an important clue for the discrimination. These participants could perceive carrier-like and speech-like sounds from SM-BCU. Even if SM-BCU, all participants could recognize the words of “hato” with the duration 220 ms at the accuracy rate of 100% and “haato” with the duration 340 ms at the accuracy rate of 95–100%. Figure 3 shows the logistic functions obtained in the behavioral tests. The threshold means for both ACAS (269.4 ms) and SM-BCU (274.6 ms) were between 260 and 280 ms. There was a significant difference between ACAS and SM-BCU thresholds (*p* < 0.05) (Figure 4a).

Figure 4b shows the boundary width. There was no significant difference in boundary width between ACAS and SM-BCU (*p* = 0.46).

## 4. Discussion

**Figure 5:**
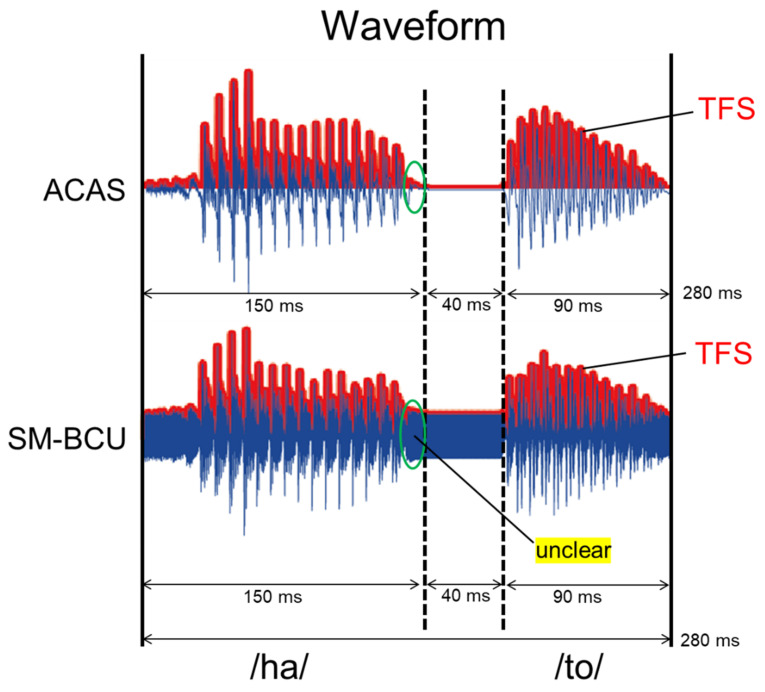
Envelope of 280 ms speech signal for ACAS and SM-BCU. ACAS, air-conducted audible sound; SM-BCU, speech-modulated bone-conducted ultrasound; TFS, temporal fine structure.

The present study investigated the categorization boundary of vowel durational changes in SM-BCU. Although there was no difference in boundary width for the categorization of “hato” and “haato,” the SM-BCU threshold was significantly longer than the ACAS threshold. These results suggest that SM-BCU can deliver prosodic information about vowel duration, and that individuals with normal hearing can categorize short- and long-duration vowels in SM-BCU with a similar difference limen to that of ACAS. The recognition of “haato” in SM-BCU required a longer-duration vowel for the categorization than in ACAS. This may be explained by the difference between SM-BCU and ACAS waveforms. Since the modulation method in this experiment was based on a double-sideband transmitted carrier, the SM-BCU waveform contained the carrier signals at the frequency of 30 kHz. The carrier signal presented consistently is a possible factor that caused the difference. Although ACAS showed silent intervals (40 ms) between the first and second syllables, the same interval in SM-BCU was occupied by the carrier signal (Figure 1). Temporal fine structure (rapid oscillations with a rate close to the central frequency of the band) plays an important role in understanding speech sounds, especially in background noise conditions [32]. Because the tail fluctuation of the envelope /ha/ in SM-BCU was unclear compared with that in ACAS (Figure 5), identification of “haato” in SM-BCU may need the longer-duration vowel. To confirm the effects of these factors, further study using other modulation methods or modulation depth is needed.

Findings from a previous study on the perceptual mechanism of SM-BCU in normal hearing individuals suggest that both demodulated low frequency sound and direct ultrasonic stimulation contribute to the recognition of SM-BCU [33]. Therefore, Future studies including a demodulated sound masking condition or examination for the performance of profoundly deaf individuals is needed on vowel durational changes in SM-BCU.

## 5. Study Limitations

This study has some limitations. We investigated word categorization of vowel durational changes for SM-BCU using only “hato” and “haato”. However, in the investigation using other vowels, duration was not confirmed. Second, the effect of order in which the measurement was performed for ACAS first and followed by the SM-BCU was not counterbalanced. Third, amount of data was relatively small. Further studies are needed to prove the consistency in other vowels and words.

In summary, through the behavioral study, the evidence for the categorization of vowel durational changes was demonstrated even for SM-BCU. This study suggests that SM-BCU can deliver prosodic information about vowel duration with a similar difference limen to that of ACAS.

## Data Availability

Not applicable.
